# Record of Meteorological Conditions at Buffalo, for January, 1855

**Published:** 1855-03

**Authors:** Sanford B. Hunt


					﻿ART. VI. — Record of Meteorological Conditions at Buffalo, for January,
1S55. By Sanford B. Hunt, M. D.
-
*•	*E
•	P	00	S	o
CO	7“	D	.	o
W	o	U3	o	O
ai	d:	o	d-	i-
<	a>	d	<a	o	j
S	t	o	*	C
S	gffl	§ S
...	<®	“5
g	ll	= a §	-g
s	C. s ;	5 £ §	£	2
a?_____co m_______________o_____________o g> ________
' •ajiuruadtudj, I	I i
jo aliuBj jsajBajg io cococicof-ooM<-cf<oo —< m — itcir-iCTinoctmcioint'O
I ■ ■	1 —	—1	— Ct__________________________________—__Ct_______________|_
‘All	X X I* L': xc? 'G ir; 7. O iG 1G H |G H C H	Irr.
-DHIintt TTT»aTA- T1 T	GD T; CT GT 07	i‘7 tc r:. c? 7? IO
' •	-• *- J '*-	i f - - ~ z~. i <’ / ~. f t. j / c*. / x cr. ex cr. cr. 'o
-lui'i t	r< 21 £2 ',' ££• 2 £2 ~	£2.	si	:n	jo	cd	r-	cd	co c:	o ci cn co ci cn co ci ci cn cd co—ib6
tuJ’d	SC Cl Gl 70 t~- Cl CO CO -C	CGiO	Cl	CG	CO	IO	<"■	OTj	CO Cl	— Cl 70 — Cl CD CO Cl — CO CO CO 11.0
<	■M8Q ubok	o c. i.n — — co td o	ci ci	ci	—‘	cd	tri	co	ci	ci -c	id od cd — o od c id ci id — <d	1
a ___________ctcocococo-^coctcoctcoco co — oici ci ct ct ct ct ct —_—■_ct — ct ^—ct lot
aJn,,J coicoco .cgcgco. . . .co	co co co oo co m m m	ifd
-aaatnanreoK	r-ooooojin oocoo cd inoo	— t^cd^oidodci^o^oi
1	—	Gt go Tf ij co co en w ct cn co—i ct ct co co st ct oo at — — — ot — ci ct ci ct 'co
12.2222-s >n o o o « «io ooomo n	o	iao
• ‘X jipiuinjj 5 2 S? £2 2? £2 £2 £2 £S 5 £2 22 2? cocococococo	co	oo
u	T-r.	11 jCD 00 00 00 Cl	00 CO Ci 00 CD 00 Cl Cl CO	00 00 00	C5	00	05	ao	joo
2	co	cd cd cn co	co co~co co co co co co	co cn co	co	co	co	cd	a£
§	-juioj	Aiaa	si co co co o	. co. co co. co co co co co	cocococococo	co	5
£	£•	£' 2	~	lo n <751- ~ d o a	ci —’ ci	rd	r<	id	ci	ci
S) ._______Ct Gt ~d CO Ct •Q* Ct Gt Gt — co — Gt— GtCOGtCtCtCO	—	Gt
• ~“ ■ ainj duia t I................ .	c2
’	12 2 i2‘2£tctciGtGt-Gocncna>	rd co -d	ct cd	-g	ct
_ -----------1 co co -o< co co m cocncoct'G'Gci—	ct co co Gt	co co	ct	ed	I
ci •----------i--:--------£—£—;-----;—.- . .	— --—.	.	—	—
S ri	. P3 — pt jG	jp i>£ fc£
g	=	fecQcaaife	^02’0202 x
<	___________si — — ctcdcdcd — — oed — rf-df	rd
5?	spnoojo j.ui y i aoocoooiouooooc co o	c o c c c o o	o	j~
5	15 £2 ~ £2 5 ~ £2 ~ — — — — ° ‘-s 'Q in - o <n o m c m co m c c c c. m m —
£	-XaTPiainn x 2	~	£2 —coccocococo-cococococococococococococnco oo
£ d	Cl 00 Cl CO Cl 00 00 00 00 S 00 Cl Cl Cl 00 00 Cl 00 <?1 00 Cl 00 Cl 00 00 00 00 Cl C71 00
2	22 5 5 5 5	cijmctocscnccna'cdTFcoXocdcd" co cn co cc i m
c • juio j aio(] de • - 5 °? .co . . .co , co co co co cocococccococococococncncccoo
£	’	di — 2 £2 22 5 £2 5 5	5	5	—	ci	m’t-cd	cd	5	rd	>n X	X	ni 5 ci — rd — cd rd — xd
_;	br--------St -G — cg CG -f CG QI CG	Gt	CG	CG	QI	—	QI	QI	CG	CO	Gt	Gt Gt	Gt	— — — Ct — CO — — Gt 'Ct
. — ■ajtij.duiaj, ........................................ .	—
a,	— 22 5 — 92 5 22 5	22	“	5	5	01	si	o	m	m	cd rd	id ci id 5 ci ci — <d
" •	•	- • 'S CO CG CO	CO	—	CG	CO	—	CO	Gt	—	CO	Gt	CO QI	Q!	— — — Gt Q) CO G’ G> QI CO
«2 W a: «2 co ai	^^0Q^i?5^^i»0Qa;^^a2«jcc«j^!25
-----------— — — co mGt< —	ct cd —'cdcdcdcdcdat — — cdt-d-o'cn — eded	-g<
spno[3jo j.aiylo o o o i.n — o —	ct o o C5 c o o oo ci o o o o o cs o cc oo	o
. r.—2 m2 2 2 E ~ 2 lf? !2coioinoinouiuoioiflwioooinio	ci
. •AjTpttunH	— 2£2 cc m'G g.g n nee n ccnn e nn	co
u	xj oi ao co jo T' ci ac ci cioaciciaocooociciciciciaioooaio	ci
a	S £2	22	2£ £2'~2	~ co’co co co co cd 'cd'co'co'co'co ' co co cd	o
. § ‘JU’Od A13Q •„•• • ■ .5 St co co . .cococococococococococo co co co	ct
2	H ’	22 2 — 5R?£2£2£222 co od -d oo co oi ud ci at Qi co id ci io ci cd	id
tc---------—<atcoTj<ctcoTt<ctco	Qteodfr at ct ct ct at ct ct ct______— — —______________ct
<i M	_	"	_	_	’	°t
•ajnj<dtnaT..............................  .	.	.	.	St
rd	!G£J2?£2z2J£2£222ee	ct oo -g — G r-i—Mmoa ooctoioct utm	od
____________Gt CO CO Tt< CO-^1 Tt< ct CO	CtCOTj<—Qt Cl CO CO QI Cl Cl QI — — Ct _________Qt
=	x	xxx^^xxxxxx'ii
___________’-'r-teor^r^O^	c6c6cQ^c6'^TjScOCQG9^iO»H»-^»—'*-*00^
spnojojo^uiy ocnncoai ooo	ooooooioooooocooooc^o	|
’ io	r- co in	o co	o>
U	-uroit io -in	go i-	co v>	o cd	m g
rH	UV«UJU U]	(7Q	QJ	o i-;	*
5 ---------------—-_______1_________1__I__________1__=5 ’_________________loo _
I	-----------------
G -uiojtsn io uil	*-2 .5‘S.	. . co . c> oo . ct oo ci . p~.	co ct qi ci m c-io	o
•*1	ci ci	ci ci ci	ci ci ci ci od ao	ci ci oo od ci oo’	co od ci oo od oc co co' ci ci	ci
__________ I	_C’	Q'	st	ci	ci	ci	c> ci ci ci ci ci ci ci ci ci ct ct ct ct ct ct ct ct ct ct ot	ct
' ct	70	T	I.G	m	t'.	CD	Ci O — Cl CO C< ia 50 l- OC Ci O — Ql CO — in CO 00 Cl o —	'
___________d^CTI_______________— -<—.—1 — — —. — — — QtCtCtCtatCtCtCtCtCtCGCO
*Amount unknown owing to drifting. . Feb. 12, depth of snow 20 inches, making of water
4 196.
				

